# Trigeminal Neuralgia, Glossopharyngeal Neuralgia, and Myofascial Pain Dysfunction Syndrome: An Update

**DOI:** 10.1155/2017/7438326

**Published:** 2017-07-30

**Authors:** Mohammad Khan, Shamima Easmin Nishi, Siti Nazihahasma Hassan, Md. Asiful Islam, Siew Hua Gan

**Affiliations:** ^1^Community Medicine, School of Dental Sciences, Universiti Sains Malaysia, 16150 Kubang Kerian, Kelantan, Malaysia; ^2^Orthodontic Unit, School of Dental Sciences, Universiti Sains Malaysia, 16150 Kubang Kerian, Kelantan, Malaysia; ^3^Hematology, School of Dental Sciences, Universiti Sains Malaysia, 16150 Kubang Kerian, Kelantan, Malaysia; ^4^Human Genome Centre, School of Medical Sciences, Universiti Sains Malaysia, 16150 Kubang Kerian, Kelantan, Malaysia

## Abstract

Neuropathic pain is a common phenomenon that affects millions of people worldwide. Maxillofacial structures consist of various tissues that receive frequent stimulation during food digestion. The unique functions (masticatory process and facial expression) of the maxillofacial structure require the exquisite organization of both the peripheral and central nervous systems. Neuralgia is painful paroxysmal disorder of the head-neck region characterized by some commonly shared features such as the unilateral pain, transience and recurrence of attacks, and superficial and shock-like pain at a trigger point. These types of pain can be experienced after nerve injury or as a part of diseases that affect peripheral and central nerve function, or they can be psychological. Since the trigeminal and glossopharyngeal nerves innervate the oral structure, trigeminal and glossopharyngeal neuralgia are the most common syndromes following myofascial pain dysfunction syndrome. Nevertheless, misdiagnoses are common. The aim of this review is to discuss the currently available diagnostic procedures and treatment options for trigeminal neuralgia, glossopharyngeal neuralgia, and myofascial pain dysfunction syndrome.

## 1. Introduction

Neuralgia is known as pain that occurs in the nerve pathways. Usually, neuralgia is not a sickness but a symptom of an injury or a disorder. Pain in the maxillofacial region exhibits medical, dental, social, and psychological burdens. Maxillofacial pain originates from many sole target tissues such as the meninges, cornea, tooth pulp, oral/nasal mucosa, and temporomandibular joint, thus showing numerous unique physiological features associated with the spinal nociceptive system [1]. Maxillofacial pain disorders cover a major remarkable and extensive part of neurological disorders and collectively have a high occurrence rate and an often overwhelming influence on the quality of life [2].

Although there are several common features of pain transduction and processing between the trigeminal and spinal nerve systems, numerous characteristic features in the peripheral and central components of the trigeminal pain system exist. Trigeminal neuralgia (TN) is the incidence of uncontrollable and electrical stun-like pain with a trigger zone, while glossopharyngeal neuralgia (GPN) is considered as pain in the oropharyngeal area throughout the mandibular actions, mainly deglutition [3, 4]. Myofascial pain dysfunction syndrome (MPDS) is categorized by extensive pain, decreased pain relief, sleep disruption, exhaustion, psychosomatic distress, and chronic headache. Patients with MPDS are identified based on the presence of numerous tender points [5]. Consequently, current basic and clinical studies that focus on acute and chronic maxillofacial pain need to realize the unique features of the pain system and to advance and assess better treatments for orofacial pain.

The most important obstruction of enhanced patient care and translational research is the absence of approved diagnostic criteria [6]. The intricate innervation and function of facial region assemblies make the diagnosis of facial pain and its treatment very problematic and frustrating [7]. Patients with long-lasting facial pain should be cautiously reevaluated and clinically examined even after getting multiple treatments. The situation is compounded when neuralgia, ear nose and throat (ENT) diseases, dental pain, myofascial pain syndromes, tumors, temporomandibular disorders (TMD), neurovascular pain, or psychiatric diseases commonly present with covering signs and symptoms. In addition, referred, severe, and acute pain frequently makes the diagnosis more challenging [7, 8]. However, most neuralgia conditions demonstrate similar clinical features. Therefore, sequential clinical examinations with additional laboratory diagnoses are essential for the proper treatment and management of such conditions [9].

An effective evidence-based management of neuralgic pain requires a thorough appreciation of the underlying mechanism of pain. In this review, we describe the updated knowledge on the pathophysiology, clinical features, diagnostic criteria, and management of TN, GPN, and MPDS.

## 2. Trigeminal Neuralgia

Trigeminal neuralgia is a unilateral disorder highlighted by electric stun-like neuropathic pain near the distribution of the trigeminal nerves with sudden onset and termination. It is defined as a syndrome that is characterized by paroxysmal facial pain. According to Loh et al. (1998), the affliction site ratio was observed as 1.4 (right) : 1 (left) of the facial area [10]. The characteristics of TN include sudden, severe, periodic, stabbing, lancinating, lightning-like, and shock-like pain attacks that are usually one-sided in the 2nd and/or 3rd trigeminal branch [11–13]. TN is one of the most common neurological pains involving the orofacial region, which generally has the most intensive type of pain [4, 14, 15]. It typically affects the elderly (1 in 25,000 of the population), with the most frequently reported cause being neurovascular compression [16–18]. From an etiological aspect, TN is classified as classic or primary or idiopathic and symptomatic or secondary. Classic or primary TN appears with no clear cause. Symptomatic or secondary TN occurs with the presence of intracranial lesions such as a tumor, infarction, and multiple sclerosis (MS). From a symptomatic aspect, TN is classified as “typical” in the presence of paroxysmal pain alone and “atypical” when the paroxysmal pain is associated with constant pain [19].

Pain in 95% of the TN cases is almost unilateral, commonly affecting the mandibular and maxillary divisions [13]. According to Loh et al. (1998), there was a greater contribution of the mandibular branch of the trigeminal nerve than the maxillary branch in TN patients (*n* = 44) [10]. An attack of TN is typically originated by a slight mucocutaneous incentive in the region of the affected trigeminal nerve also known as a trigger point [18, 19]. The diagnosis of TN is based on the typical case history of episodic electric pain in the supply of the trigeminal nerve [20]. However, the diagnosis and management of TN require a group of experts consisting of neurologists, anesthesiologists, neurosurgeons, neuroradiologists, oral and maxillofacial surgeons, and dentists [21, 22].

### 2.1. Epidemiology

The prevalence of TN has been estimated at 107.5 males and 200.2 females per 1 million populations [23]. The incidence rate of TN was 4.3 per 100,000 in the US population, with the age-adjusted rate for females being significantly higher than that for males [24]. The predictable frequency of TN is approximately 4–12.5/100,000 people annually, with increasing occurrence based on stage [25]. Because age is a primary risk factor, symptom manifestation is more likely after the age of 50 years [26]. The topmost inception of TN occurs between the ages of 50 and 70 years, but it can happen even at a younger age. A study reported the usual age of sign onset at 19.6 ± 3.4 years [27]. The most widely recognized age group was 51 to 60 years (36.90%), followed by 61 to 70 years (23.68%) and 41 to 50 years (17.35%) [28]. It is unusual in individuals older than 30 years, with only 1% of cases in those more youthful than 20 years of age [29, 30]. The annual occurrence in females is approximately 5.9/100,000 cases per females, while it is approximately 3.4/100,000 cases per males [25, 31]. The female-to-male proportion of TN was 3 : 2 [32]. TN is more common among females (62%) than among males (38%), with a female-to-male ratio of 1.6 : 1 [33]. Thus, females have a higher risk of having TN than males, and the risk again increases with age [30].

In addition, a retrospective study of TN patients in Singapore and Malaysia revealed that the age of onset fluctuates from 24 to 89 years with a mean age of 54.9 years. The ultimate occurrence was in the 6th (29.5%) and 7th (27.3%) decades of life, followed by the 5th decade (13.6%). The fourth and eighth decades had similar incidences (11.4%). This was followed by the third (4.5%) and ninth (2.3%) decades. With respect to sexual orientation, females account for 63.7% of the patients, with a ratio of 1.75 : 1.00. The distribution of TN in multiethnic populations was 68.2% among the Chinese, followed by 13.6% and 11.4% in Malays and Indians, respectively. The remaining distribution was solitary Japanese and two Eurasians [10].

Other than the age and gender, multiple sclerosis (MS) is a well-known risk factor to TN [10, 27]. MS has been reported in 2 to 4% of patients with TN where demyelination is also present [24]. The reported prevalence of TN in the MS population was between 1.0% and 6.3% [34, 35]. In addition, approximately 4% of MS patients have a lifetime risk to have TN, with no significant difference observed among the different forms of MS [36]. Approximately 5% of idiopathic TN cases have been reported to have a family history [33]. Hence, older females, MS, and family history are important risk factors for TN.

### 2.2. Pathophysiology

TN occurs due to the specific abnormalities of the trigeminal nerve in the trigeminal root or ganglion. The pathophysiological characteristics of classic or idiopathic TN are identified with the pressure of the trigeminal nerve root by a vein at or nearby the root passage zone [17, 37, 38]. An artery crossing the nerve can provoke further displacement [39], which can lead to damage and injury of the trigeminal nerve. The damage tends to be localized and is specifically related to the vascular contact [40, 41]. The damaged nerves cause pain via several mechanisms, including the hyperexcitability of the demyelinated nerve fibers, ectopic impulse discharge, spontaneous and triggered after discharge, cross excitation between sensory channels, deafferentation, impaired segmental inhibition, and emphatic transmission [38, 40–43]. The vascular pressure of the trigeminal nerve root is associated with the focal loss of myelin and the close juxtaposition of the demyelinated axons with few mediating astrocytic processes [40].

Symptomatic or secondary TN occurs due to intracranial lesions such as a tumor, infarction, and MS [19, 44]. Intracranial tumors in the presence of aneurysms, angiomas, or vascular malformation are among other causes of TN [45, 46] that may occur by either direct tumoral compression or the wrapping of the trigeminal nerve root [47, 48]. Tumor occurrence has been reported to be more common among TN patients aged less than 39 years than among those older than 40 years [49]. Approximately 2 to 4% of MS has been reported to occur in TN patients [24] as well.

### 2.3. Clinical Manifestation

The hallmark of TN includes recurrent attacks of lancinating pain in the trigeminal nerve [50]. The attacks typically last just seconds, yet they may be repeated over and again inside a brief time frame. Approximately 79% of the pain experienced is intermittent, while 21% of pain is continuous [13]. The nature of pain is sporadic, sudden, and often like an electric shock, enduring from a few moments to a few minutes [27]. In fact, TN has been reported to be a relatively common pain condition, occurring more commonly on the right side (72.63%) than on the left side (27.37%) with a ratio of 2.6 : 1.0 [30, 51] and mainly affecting the mandibular division [10, 30]. The communal outlying nerve involved is infraorbital with various patients presented with sensible pain [30].

The brief paroxysms pain in TN is restricted to the facial conveyance of the trigeminal nerve and can be triggered by stimuli to sensory endings in the trigeminal receptive area [52]. The triggering stimuli include simple actions such as talking, swallowing, laughing, washing, wind-blowing, shaving, mouth opening, touching, and chewing. Nevertheless, the presence of these triggering stimuli may cause the patients to circumvent any stimulus on the face or mouth. Most patients respond to different strengths of the aggravating stimulus and react to more than a single triggering stimulus [53].

### 2.4. Diagnostic Criteria

**Figure 1:**
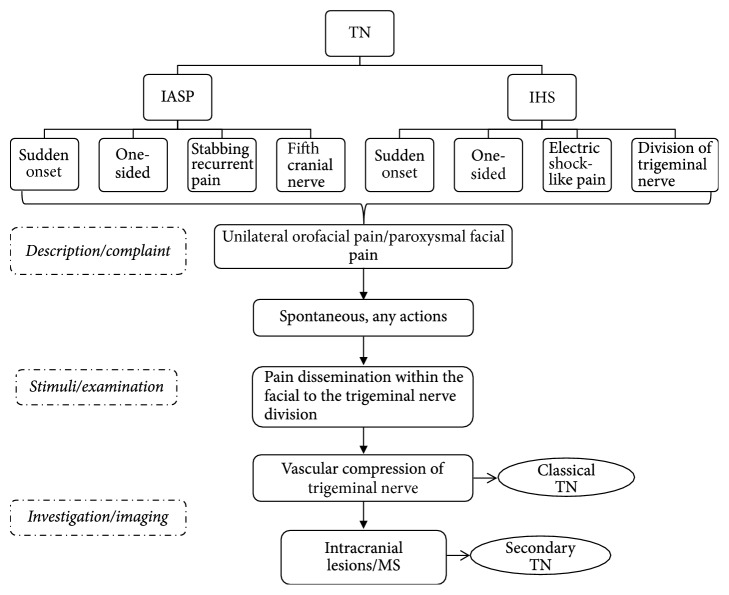
The basic diagnostic criteria of trigeminal neuralgia (TN).

The diagnosis of TN basically relies on a patient's description of pathognomonic pain occurrences [54]. The primary diagnostic tools are MRI (magnetic resonance imaging) and CT (computed tomography), since there is no specific laboratory test available (Table 4). Therefore, patients' signs and symptoms are key important factors in making the diagnosis (Figure 1).

TN is generally idiopathic [55]; however, it might likewise emerge optional to different conditions, including the intracranial space involving lesions and multiple sclerosis [35, 56, 57]. The International Association for the Study of Pain (IASP) and International Headache Society (IHS) has recommended their own indicative criteria for TN [58, 59]. The IASP has defined the TN as a sudden, normally one-sided, extremely brief cutting intermittent pain in the dissemination of at least one branch of the fifth cranial nerve. On the other hand, the IHS defines TN as an excruciating one-sided distress of the face, described by brief electric stun-like pain restricted to the circulation of at least one division of the trigeminal nerve. Pain is usually induced by insignificant stimuli, which include smoking, sneezing, and brushing the teeth, all of which often regularly occur impulsively. Little zones in the nasolabial fold and chin might be prone to the precipitation of pain that may remit for variable periods [58].

According to Eller et al. (2005), TN is defined and classified into seven types: (i) TN1: idiopathic, sharp, shooting, electrical shock-like, and episodic pain; (ii) TN2: idiopathic, aching, throbbing, burning, and more than 50% constant pain; (iii) TN3: trigeminal neuropathic pain and accidental harm to the trigeminal nerve from injury or surgery (facial trauma; oral operation; ear, nose, and throat operation; skull base operation; posterior fossa operation; or stroke); (iv) TN4: trigeminal deafferentation pain and intentional injury to the trigeminal nerve (gangliolysis, nucleotomy, neurectomy, tractotomy, rhizotomy, or other denervating procedures); (v) TN5: symptomatic, associated with MS; (vi) TN6: postherpetic because of a flare-up of facial herpes zoster; and (vii) TN7: atypical facial pain and facial pain auxiliary to a somatoform pain issue, requiring mental testing for indicative diagnostic affirmation [32].

### 2.5. Treatment and Management

Table 1 presents various pharmacological agents and available treatment options based on surgical procedures. Both methods are effective and widely used [60–62]. Usually, TN patients are first treated with pharmacological agents. The pain can be readily managed with medication in approximately 80% of patients [63]. However, if the pain is not effectively relieved by medications or there is medication intolerance due to toxicity or allergic reactions, surgical treatment becomes an option.

In standard practice, the first-line treatment is carbamazepine, which can relieve most of the observed symptoms [22, 64–66]. Other drugs, including oxcarbazepine [67], phenytoin [68, 69], baclofen [70, 71], lamotrigine [72, 73], gabapentin [74, 75], and sodium valproate [76, 77], are also efficient in reducing the signs-symptoms of TN in most patients. Sometimes, some of the drugs are taken with carbamazepine as an adjuvant for the synergistic effects in relieving the TN symptoms [78, 79]. The decreasing relief provided by carbamazepine or other drugs with continual use as well as unacceptable side effect profiles may necessitate the discontinuation of therapy. In fact, it has been reported that approximately 50% of patients eventually require an operation to relieve pain [25]. Research has indicated that there is no significant influence of age, sex, ethnicity, or the side of the face on the decision of the medication regimen and the length of treatment for pain control [79].

When drugs no longer offer pain relief, surgical intervention is preferred. The objective of surgery procedures is to prevent the blood vessel from squeezing the trigeminal nerve or injuring the trigeminal nerve. Several surgical approaches used to relieve the pain due to TN include neurectomy of trigeminal nerve branches outside the skull, percutaneous radiofrequency thermal rhizotomy, percutaneous ablation that creates trigeminal nerve or trigeminal ganglion lesions with heat [80–82], percutaneous retrogasserian glycerol rhizotomy, injection of glycerol into the trigeminal cistern [62, 83, 84], physical compression, trigeminal ganglion balloon microcompression [85], alcohol injections [57, 86], botulinum toxin injection [87, 88], cryotherapy [89, 90], and gamma-knife radiosurgery (GKRS) [91]. These procedures are intended to alleviate the symptoms of TN by relieving nerve compression at some point along its course. Hereafter, the management of TN is dependent on a fast, correct diagnosis, prompt, and effective treatment because the symptoms can be very severe.

### 2.6. Complications

Many drugs used in the treatment and management of TN are associated with several side-effects. Nevertheless, most of the effects can be tolerated and immediate drug discontinuation is conducted when the common pain tolerance level is exceeded. In addition, some of the surgical procedures may contribute to some complications such as microvascular decompression (MVD) which is able to relieve an abnormal compression of an artery to the trigeminal nerve. Although it is generally successful, achieving 70–90% pain control and less than 1% of the mortality risk [92], the procedure may lead to hearing loss which is associated with a refutation injury of cranial nerve VIII [93]. Nevertheless, the complications were less common following an intraoperative monitoring of the brainstem evoked response.

The rate of ipsilateral hearing loss was 3% (previous 1980) and 1% thereafter (*p* = 0.008). Radiosurgery, which can increase the pain control rate up to 83%, is effective in treating TN [91]. Its complication rate is only 6% for facial paresthesia and 4% for hypoesthesia [94]. An immediate complete loss of vision in one eye after trigeminal radiofrequency rhizotomy was reported in three patients due to acute traumatic optic neuropathy [95]. Moreover, there were patients who have masseter weakness and paralysis, keratitis and transient paralysis of cranial nerves III and VI, diminished corneal reflex, dysesthesia, and anesthesia dolorosa [96]. Nevertheless, MVD remains the best choice and a useful alternative to carbamazepine in the treatment of TN (Table 1).

## 3. Myofascial Pain Dysfunction Syndrome

Myofascial pain dysfunction syndrome (MPDS) is known as a psychophysiological disease, which is associated with muscular structure—in particular, the muscle of mastication [97]. MPDS is a commonly observed phenomenon in the medical, dental, and psychological sectors. MPDS is also termed myalgia, myofasciitis, myogelosis, myofascial pain, fibromyalgia, myofibrositis, and myofascial pain syndrome (MPS). When it is mainly associated with temporomandibular join dysfunction, it is termed as myofascial pain dysfunction syndrome [98–100]. Although the terms myofascial and musculoskeletal pain are used frequently and alternately, they are different. Basically, musculoskeletal pain encompasses almost all types of pain observed at the muscular level, while myofascial pain indicates a specific syndrome caused by the presence of trigger points (TrPs) within the muscles or their fascia [101].

The following factors play a significant role in the etiology of MPDS: (1) trauma, which is divided into macro- and microtraumas; the former includes contusions, sprains, and strains, all of which may result in acute MPDS; the latter, however, has a slower onset. Chronic repetitive overloading or the further overuse of muscles may cause fatigue; (2) mechanical causes, which can be a result of internal factors such as having incorrect postures or external factors such as having poor ergonomics, especially at work; (3) degenerative causes due to aging where the degeneration of bones and joints, loss of myofascial flexibility, and arthritis can occur; (4) nerve root compression, which may cause irritation and lead to sensitization to the spinal segment; (5) dental causes, which include occlusal disharmony; (6) faulty dentures; (7) dental extraction; and (8) other factors.

According to Svensson and Graven-Nielsen, 2001 [102], MPDS is defined as a stress-related disorder. It is assumed that increased muscle tension, commonly combined with the existence of some parafunctional habits (such as clenching or grinding of teeth), results in muscle fatigue and spasms highly responsible for the pain experienced as well as the mandibular dysfunction. However, it may also occur as a consequence of muscular overextension, muscle overcontraction or trauma in some rare cases [103, 104].

Myofascial pain syndrome is defined as a disorder that is associated with pain arising from TrPs within the myofascial structures, the pain appearing either locally or distant from the affected region. On the other hand, TrPs are sensitive areas in a muscle that continuously (or upon compression) cause pain to a distant region [99, 105]. Alternatively, it can also be described as a confined tender area in a firm band of skeletal muscle, tendon, or ligament. These points can arise in any skeletal muscle, occurring most repeatedly in the head and neck, shoulders, and lower back [99].

### 3.1. Epidemiology

MPDS appears with muscular pain and regional symptoms [106, 107] with a 30% percent occurrence, thus contributing to considerable disability and inability to work [108, 109]. It has been reported that, in the American population, approximately 44 million people have myofascial pain-associated problems [110]. A statement from specialized clinics for head and neck pain stated that 55% of cases are related to myofascial etiology, while 95% of cases have myofascial pain [111]. According to the same study (*n* = 164), 55% of patients diagnosed with myofascial pain also have chronic head and neck pain for a minimum of six months [112]. A Danish researcher reported the existence of myofascial pain in 37% of men and 65% of women within a randomly selected population (*n* = 1504) aged between 30 and 60 years [113]. The Myofascial Pain Management Center at the University of Miami School of Medicine conducted a study in an American population (*n* = 283) and reported that 85% of the cases primarily presented with MFP syndrome [114]. Another study stated that 74% of the patients had pain due to a primary cause (such as mechanical cause), while 93% complained of pain during the diagnosis. The data indicate that a high proportion of patients are unaware of their disease, with 74% of patients attending the clinic with pain that is purported to be caused by either mechanical injury or trauma [112].

Another study reported that females tend to experience a higher recurrence rate of MPDS than males, with the male : female proportion ranging from 3 : 1 to 5 : 1 in various reports [115]. The age group showing the highest incidence was the 20- to 40-year age group, although children can also have MPD syndrome [116]. The mean prevalence of pain among middle-aged to advanced-age individuals (30–60 years) is 37% for males and 65% for females. In advanced-aged patients (>65 years), the prevalence can be as high as 85%. As a result, based on the demographics of aging, MPS can most likely convert to one of the major problems in the general population in the future [101].

### 3.2. Pathophysiology

The origin of TrPs in MFP syndrome is still unknown, although there has been significant progress in the identification of the features of TrPs [117, 118]. Precipitating factors of MFP (such as nerve root compression and degenerative cause) have been hypothesized to lead to the discharge of acetylcholine at motor end plates [119], thus causing muscle fiber contractions. The release of vascular and neuroactive substances causes local ischemia and also aggravates muscular pain. Further acetylcholine discharge can propagate the muscle pain and spasm. After a prolonged period of time, local muscle fibrosis can occur [119, 120]. The myofascial trigger point (MTrP) contains a neurovascular bundle that consists of motor nerve endings and groups III and IV nociceptive sensory afferent nerve endings [121, 122]. Triggering the nerve can lead to the sensory and motor phenomena. In sensory phenomena, examined the nociceptive process in myofascial TrP pain in chronic tension-type headaches with 40 cases compared with 40 healthy controls. The pressure in MTrP showed a linear relationship with incremental pain, while the normal muscle tended to show a nonlinear relationship. Some mechanisms in the spinal cord that are associated with dysfunctional endplates in response to sensitized sensory nerve fibers may also be involved. The linear pattern of pain indicates that referred pain may occur in response to stimulation of MTrP compared with the normal muscle [123]. On the other hand, in motor phenomena, pathophysiological marker-needle electromyography (EMG) activity has been identified within the 1 to 2 mm nidus of MTrP. In 1986, Laskin and Block hypothesized that the source of electrical activity was intrafusal fibers inside the muscle spindle [103]. However, Greene proposed a standard hypothesis about dysfunctional motor endplates in 2001. According to them, such electrical activity is demonstrated in the endplate zone outside of MTrP [97].

### 3.3. Clinical Manifestation

**Figure 2:**
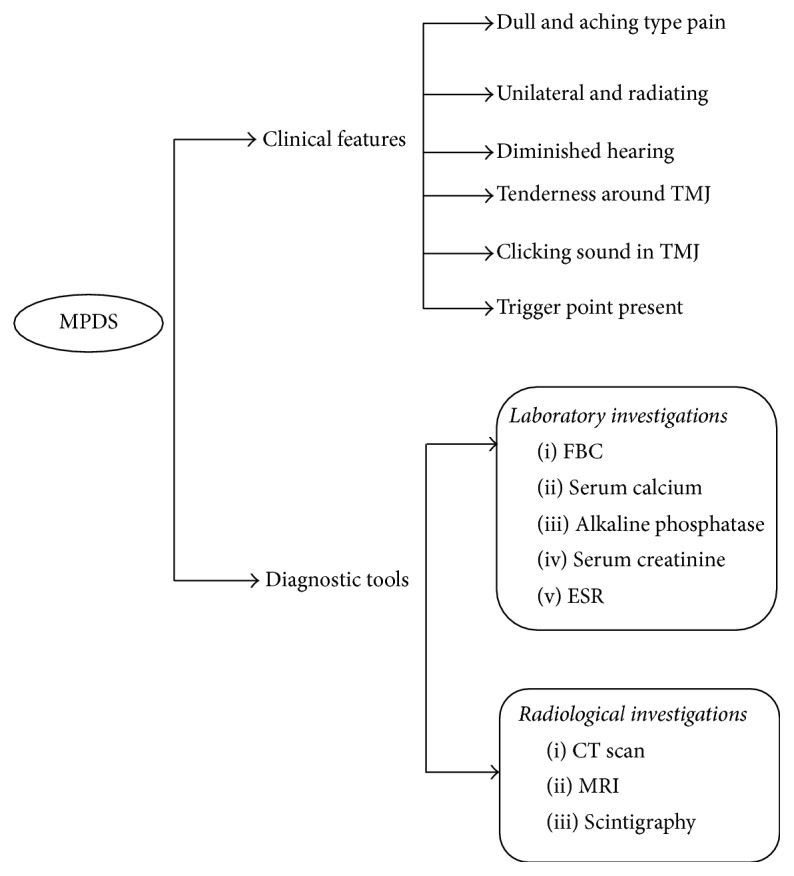
Commonly used diagnostic tools and features of myofascial pain dysfunction syndrome (MPDS).

MPDS is characterized by a dull, aching, radiating pain and is more acute in jaw movement (Figure 2). In the case of mandibular dysfunction, MPDS causes limitation in mouth opening [124, 125]. Almost 70% of patients complain of pain and tenderness around their temporomandibular joints. Some patients also reported the presence of a clicking sound or cracking noises in the temporomandibular joint (TMJ) [124]. MPDS usually involves only the face unilaterally. Frequent headache is also a common complaint (23% of cases). Only tension headaches may be directly or indirectly associated with this syndrome. In addition, a patient may also complain of diminished hearing, tinnitus, burning tongue, and neuralgic pains. MPDS also leads to some functional disorders and may cause organic variations in the temporomandibular joint as well as in masticatory muscles [103, 125]. Usually, the patient shows pain in the trigger area when approximately 3 kg of pressure is applied. The fibers show the local twitch response (LTR) during plucking palpation inside the band of muscle [107].

### 3.4. Diagnostic Criteria

Myofascial pain syndrome is associated with MTrPs and can be reproduced by firm palpation over the trigger zone. The diagnostic features for myofascial trigger points include the following:

- Upon palpation of the muscle, a focal point of tenderness may be found.
- Trigger point palpation leads to a reproduction of the pain.
- There is a “taut band” in adjacent muscle.
- There is restriction of muscle movement.
- It may appear with pseudo-weakness of the muscle fiber.
- Paresthesia occurs over the muscle area.

The diagnosis of MFP syndrome may be a challenge because it shows some complex clinical characteristics. Most patients tend to show some tenderness (67%) of the elevator muscles during palpation. Nearly 40% of the patients' complain of pain on chewing. Thus, 30% have significant myalgia with bruxism [126]. Sometimes, the patients may also complain of sleep disturbance because the sleeping position may act as an aggravating factor to the pain caused by MTrP [107].

Arthrography is a useful procedure for the diagnosis of MPDS when the TMJ is involved. Radiographic interpretations of the head and neck region, CT scans and scintigraphy, can also help to establish the concluding diagnosis. In this case, MRI is indicated to allow better view of the condyle and disc position of TMJ specifically. Sometimes laboratory tests such as a complete blood count (in the case of suspected infection), serum calcium, phosphorus, and alkaline phosphatase (for bone disorder) need to be investigated. Serum uric acid (for gout), serum creatinine, and phosphokinase levels help as useful indicators of muscular disorders. If rheumatoid arthritis is suspected, the electrolyte sedimentation rate (ESR) and rheumatoid factors should be investigated. Additionally, electromyography can be used to assess muscle function and fatigue. However, psychologic evaluation and psychometric testing may also help in the diagnosis [127].

### 3.5. Treatment and Management

Patient counseling regarding the disease is very important. Usually, it is often difficult for patients to accept a psychophysiologic explanation for their diseases. Connecting the symptoms to specific masticatory muscles can facilitate the determination of the actual cause of the type and location of pain. In cases of the involvement of the temporal muscle, patients may suffer where headache and jaw ache are common in the masseter and pterygoid muscles, showing discomfort during swallowing and earache associated with the lateral pterygoid muscle [128].

Normally, injection is the first treatment approach in the treatment of MPDS. An injection using dry needling and lidocaine anesthetic solution injection may be helpful in MPDS treatment. In 2010, Ay et al. [129] reported that using dry needling and lidocaine injection with stretching exercises may show a momentous role in the treatment of MFP syndrome. Besides, botulinum toxin injection has been reported to produce significant effects such as sudden pain reduction, especially when a saline injection is not effective [130–132]. Moreover, Gazi et al. (2010) compared the analgesic result of acupuncture (which is purported to help restore blood circulation) to TrPs injections (containing 0.25% bupivacaine administered twice weekly) with cyclobenzaprine chlorhydrate and sodium dipyrone on trigger points to prevent the recurrence of MFP symptoms. The results showed comparable pain liberation and progress in the quality of life at 4 weeks [133]. Additionally, therapeutic ultrasound is a new treatment strategy for MFP syndrome. Because it is a noninvasive procedure, it is valuable in the treatment of deeper muscle. Manual therapeutic techniques are not sufficient for the assessment of deep muscle [134]. Other approaches have been used in the treatment and management of MPDS including psychotherapy [135, 136], home therapies [137], medications [138, 139], and dental management [140, 141]. Sequential treatment is needed to manage such pain syndrome (Table 2).

### 3.6. Complications

In the case of a misdiagnosis, chronic pain syndrome and complex behavioral problems may result, leading to psychosocial problems. Most of the time, chronic pain may cause sleep disturbance where patients have trouble finding a comfortable sleeping position. Sometimes, posture changes during sleep may affect the disease, thus affecting the patients' sleep throughout the night [108].

## 4. Glossopharyngeal Neuralgia

Glossopharyngeal neuralgia (GPN) is a very sporadic condition related to hyperactivity of the glossopharyngeal nerve [142]. GPN is rare compared with TN. The pain affects the sensory areas corresponding to the glossopharyngeal neuralgia with a branch of sensory vagus nerves. GPN consists of spasmodic, momentary, and severe sharp pain in the posterior area of the throat, tonsillar fossa, base of the tongue, ear canal, and areas inferior to the angle of the mandible [143]. Generally, the pain persists for seconds to minutes and is often triggered by chewing, coughing, yawning, talking, and swallowing [144]. The prevalence of GPN is estimated to be approximately 0.8 per 100,000 populations in a year, indicating that it is less common than TN (4.7 cases per 100,00) [26]. GPN is usually represented by a painful condition on the left side of the body in females, whereas TN is more commonly observed on the right side [145].

GPN is a mixture of cranial nerves that have somatic sensory fibers from the oropharynx, mastoid, middle ear, and Eustachian tube, and posterior third of the tongue. The middle ear and mastoid have a sensory supply of glossopharyngeal nerve along with the tympanic branch or Jacobson's nerve [146]. It also receives special sensory fibers for taste as well as chemoreceptor and baroreceptor afferent input from the carotid body and carotid sinus. Stylopharyngeus muscles are supplied by the motor component, and the parotid gland is supplied by the parasympathetic secretomotor supply. The nerve of Hering is an important branch of the carotid sinus branch, which conveys chemoreceptor and baroreceptor information centrally for circulatory reflux function and may be accountable for the arrhythmogenicity of GPN [146, 147].

GPN is classified according to HIS, based on the involved structural areas. The proposed classification of IHS is (1) classical GPN (occasional or episodic pain) and (2) symptomatic GPN (constant pain) [58]. The classification is based on the areas involved in otitis, that is, pain in (or around) the auricle or earlobe. On the other hand, for the oropharyngeal type, the pain is in and around the neck as well as the maxillofacial regions. Nevertheless, these areas significantly overlap with other cranial nerve-supplied areas [148].

GPN may be idiopathic with the absence of any obvious lesion. Most cases are mainly recognized as glossopharyngeal nerve compression triggered by a vessel at the root entry zone of the brainstem [148, 149]. Idiopathic causes may be vascular decompression and/or central pontine dysfunction. The secondary cause is a noticeable lesion that includes trauma (skull base fracture, penetrating injury), postradiation, neoplasm (skull base, cerebellopontine, brainstem, pharynx, tongue, tonsil, metastatic head, and neck tumors), infection (tonsillitis, pharyngitis, petrositis, arachnoiditis, para pharyngeal abscess, and tuberculosis), surgery (posttonsillectomy, postneck dissection, and postcraniotomy), vascular malformations (arteriovenous malformation, fusiform aneurisms, persistent hypoglossal artery, and dissection of the vertebral artery), demyelination (MS), and Eagle's syndrome as well as others which include direct carotid puncture, choroid plexus overgrowth, and hyperactive dysfunction syndrome. This type of GPN is usually accompanied with numbness or pain around the affected area [150].

### 4.1. Clinical Manifestation

There are paroxysmal attacks of facial pain, which last for seconds to minutes (Table 3). The characteristics of pain are unilateral sharp, stabbing, and severe shooting pain. The distribution of pain is persistent within the posterior part of the tongue, pharynx to inner angle of the lower jaw, and tonsillar fossa [151]. Pain occurs within a certain interval with most occurring after a long time, although some patients may experience it within a day [152, 153].

The common trigger factors are swallowing, chewing, talking, coughing, yawning, lateral movement of the jaw, cleaning of the throat, tinnitus, sudden movement of the head, touching the periodontium, and touching the external surface of the ear. GPN is not attributed to other disorders but may be related to vascular malformation, oropharyngeal tumor, cerebellopontine angle masses, Chiari type I disorder, multiple sclerosis, or TN [144, 154–158]. GPN may be not found during an early screening or examination [159]. In fact, GPN is often confused with Jacobson's neuralgia during clinical examination [160].

### 4.2. Diagnostic Criteria

GPN should be diagnosed by clinical examinations. Patients usually experience unilateral stabbing pain in the throat. The characteristics of pain and trigger factors are helpful to confirm GPN. Neuralgic pain is severe, episodic, and radiating for intervals compared with inflammatory pain. Inflammatory pain is persistent in nature, lasting for minutes. The distribution of pain should be determined to ascertain the involvement of the glossopharyngeal nerve or its association with other cranial nerves.

Classical GPN is mainly tympanic or posttonsillar pain with a history of surgery. Distribution of the trigger point is within the auricular, oropharyngeal area or can be triggered by swallowing, talking or hearing. Lignocaine (2%) or bupivacaine (0.5%) injection into the area close to the trigger point may be helpful for the identification of otologic pain. A complete history including trauma, radiotherapy, postsurgery, inflammation or pathology related to the oral and maxillofacial areas is useful to elucidate the cause of secondary GPN [161, 162].

Laboratory test including erythrocyte sedimentation rate (ESR), serum chemistry, complete blood count, and anti-nuclear antibody is helpful to determine if infection, inflammation, and neoplastic malignancy occur. To determine if vascular compression, any malignancy, or hard tissue change has occurred, magnetic resonance angiography (MRA) and 3D CT-angiography, magnetic resonance imaging (MRI) can be useful [163, 164]. These imaging techniques may help elucidate vascular compression by identifying the origin of the posterior inferior cerebellar artery since the posterior inferior cerebellar artery makes an upward loop and compresses the supraolivary fossette [165, 166]. If the offending vessel is the anterior inferior cerebellar artery, the diagnosis of GPN is a challenge without surgery [167]. GPN secondary to Eagle's syndrome is identified by a panoramic radiograph [168].

### 4.3. Treatment and Management

Nonsurgical pharmacotherapy is the treatment of choice for most of the cases (Table 3). Failure of pharmacotherapy treatment, where the nerve is compressed, can prompt the physician to opt for surgery.

#### 4.3.1. Nonsurgical Pharmacotherapy Approach

Carbamazepine, gabapentin, and pregabalin are first-line pharmacological treatments for GPN. Vitamin B12 and a low-dose selective serotonin reuptake inhibitor such as paroxetine (20–50 mg/day) or sertraline (50–200 mg/day) are also helpful. A long-term nonsteroidal anti-inflammatory drug is not recommended as long as the activity resulting in the inflammatory process can be reduced for treating GPN [169]. Opioids can be used, although they have been reported to provide no additional benefit [170]. If pain relief cannot be achieved, different medications such as baclofen and dextromethorphan can be used. The combination of two or multiple agents is effective with physical therapy or psychological treatment [171].

(*1) Nerve Block*. A glossopharyngeal nerve block is an excellent adjunct to the pharmacological treatment of GPN for rapid pain. A nerve block can be performed using a local anesthetic agent such as lignocaine (2%) and bupivacaine (0.5%) with or without steroids, ketamine, phenol, glycerol, and alcohol. A nerve block can be performed either as an intra- or extraoral approach. The extraoral approach is preferred, as it is simpler and more comfortable to the patient [172, 173]. Difficulties in deglutition and huskiness of voice can be an unwanted consequence of a glossopharyngeal nerve block. In addition, a bilateral nerve block can cause the paralysis of the vocal cord [172, 174].

#### 4.3.2. Surgical Approach

There are several surgical procedures offered such as direct surgical neurotomies or percutaneous radiofrequency thermal rhizotomy [173], direct sectioning of the nerve in the cerebellopontine angle [175], or open trigeminal tractotomy-nucleotomy or nucleus caudalis operations. The microvascular decompression (MVD) of vascular roots and the rhizotomy of nerve roots are the best options for surgical treatment. Vascular compression is well recovered by MVD. Normally, intracranial root section is widely considered when MVD is not possible [151, 176]. Through the improvement of microsurgical and anesthesiology techniques, MVD has been established as an effective and safe treatment option for drug-resistant GPN [175]. Over 76% of the cases showed improvement with MVD [162]. Extracranial neurotomy and percutaneous radiofrequency rhizotomy are not commonly performed, and their application is limited to drug-resistant GPN. Stylectomy is usually conducted for Eagle's syndrome after ruling out the primary cause of GPN [177, 178]. Pulsed radiofrequency neurolysis, gamma-knife surgery, and stereotactic radiosurgery have also shown beneficial effects in both idiopathic and secondary GPN [148, 179].

## 5. Conclusion and Future Perspective

This review has outlined the current knowledge of the pathophysiology, diagnostic criteria, and treatment of three common neuralgic maxillofacial pain syndromes. TN, GPN, and MPDS can present with common signs. Hence, the appropriate use of definite diagnostic measures is significant to support the differential diagnosis. Severe and diffuse pain can obscure the diagnosis, possibly leading to an inappropriate finding or an incorrect diagnosis. Precise diagnostic criteria will facilitate clear diagnoses and the framing of good treatment plans for a proper therapeutic schedule. Various pharmacological and surgical treatment options have been used with varying success rates and morbidities. There is a need for further research into the management of neuralgic maxillofacial pain with appropriately designed treatment strategies. A multidisciplinary team is frequently essential for the diagnosis and management of many maxillofacial painful conditions.

## Figures and Tables

**Table 1:** Characteristics and management of trigeminal neuralgia in the maxillofacial region.

Diseases	Clinical features	Pharmacological treatments	Side effects	Surgical /local treatments	Limitation
Trigeminal neuralgia	Pain, electric shock like Pain duration, seconds Intensity, severe Localization, good Characteristics, trigger zone, diurnal Trigger points, nonnoxious stimulus	Carbamazepine	(i) Development of resistance and intolerance (ii) Dizziness (iii) Nausea (iv) Ataxia (v) Vomiting (vi) Xerostomia	Percutaneous trigeminal rhizotomy	(i) Recurrence of pain (ii) Dysesthesia (troublesome numbness) (iii) Loss of corneal reflex
		Oxcarbazepine	(i) Dizziness (ii) Fatigue (iii) Nausea (iv) Vomiting (v) Headache (vi) Acne (vii) Dry mouth (viii) Constipation	Ablative peripheral procedures (neurectomy)	(i) Less morbidity but with chance of recurrence
		Gabapentin	(i) Dizziness (ii) Fatigue (iii) weight gain (iv) Drowsiness (v) peripheral edema	Microvascular decompression	(i) Low recurrence of pain (ii) Chance of causing nerve damage (iii) Haring loss, double vision, facial numbness, or paralysis
		Pregabalin	(i) Dizziness (ii) Blurred vision (iii) Diplopia (iv) Increased appetite and subsequent weight gain (v) Euphoria, confusion, and vivid dreams (vi) Changes in libido (increase or decrease) (vii) Memory impairment (viii) Tremors (ix) Dry mouth (x) Erectile dysfunction (xi) Peripheral edema (xii) Nasopharyngitis (xiii) Increased creatinine kinase level		
		Topiramate	(i) Paresthesia (ii) Nausea (iii) Lack of concentration (iv) Diplopia (v) Nervousness and dizziness (vi) Memory impairment (vii) Speech disturbance (viii) Disturbance (ix) Diarrhea	Gamma knife radiosurgery	(i) Tenderness develops where the screws or pins were placed (ii) Hair loss where the radiation was directed (iii) Damage to surrounding tissues in the brain, caused by swelling
Trigeminal neuralgia	Pain, electric shock like Pain duration, seconds Intensity, severe Localization, good Characteristics, trigger zone, diurnal Trigger points, nonnoxious stimulus	Valproates	(i) Skin rash (ii) Hair loss (iii) Weight gain	Myotherapy	(i) Regular monitoring is not possible
		Tricyclic antidepressants (TCA)	(i) Sedation (ii) Dry mouth (iii) Blurred vision (iv) Breast enlargement in males (v) Constipation, mania (vi) Weight gain (vii) High blood glucose level	Injections	(i) As it is a painful procedure, the patient feels uncomfortable during injection
		Sedatives (on condition)	(i) Apnea (ii) Physical dependence (iii) Decreased mean arterial pressure (iv) Tachycardia (v) Paradoxical excitement	Acupuncture	(i) Painful procedure
				Therapeutic ultrasound	(i) A skilled person is needed, and the patient is still under observation

**Table 2:** Characteristics and management of myofascial pain dysfunction syndrome in the maxillofacial region.

Diseases	Clinical features	Pharmacological treatments	Side effects	Surgical/local treatments	Limitation
Myofascial pain dysfunction syndrome	Pain, dull and aching type Pain duration, constant Intensity, moderate Localization, diffuse Characteristics, generalized, spontaneous Trigger point, palpation, function	Nonsteroidal anti-inflammatory drug	(i) Indigestion (ii) Stomach upset (iii) Stomach bleeding (iv) Peptic ulcer (v) Fluid retention	Psychotherapy	(i) Failure of episodic counseling and patient's ignorance
		Tricyclic antidepressants (TCA)	(As stated in Table 1)	Myotherapy	(i) Regular monitoring is not possible
				Injections	(i) As it is a painful procedure, the patient feels uncomfortable during injection
		Sedatives (on condition)	(i) Apnea (ii) Decreased mean arterial pressure (iii) Tachycardia (iv) Paradoxical excitement	Acupuncture	(i) Painful procedure
				Therapeutic ultrasound	(i) A skilled person is needed, and the patient is still under observation

**Table 3:** Characteristics and management of glossopharyngeal neuralgia in the maxillofacial region.

Diseases	Clinical features	Pharmacological treatments	Surgical /local treatments	Limitation
Glossopharyngeal neuralgia	Pain, dull type Pain duration, short duration Intensity, mild to moderate Localization, diffuse Characteristics, usually pain in the throat/ mouth floor Trigger point, swallowing	Carbamazepine	GN nerve block	(i) Chance of trauma to the internal jugular vein and carotid artery (ii) Hematoma formation
		Gabapentin pregabalin	Myotherapy Percutaneous radiofrequency thermal rhizotomy	(i) Regular monitoring is not possible (ii) Recurrence (iii) Hoarseness of voice (iv) Vocal cord paralysis, and dysphagia (difficulty in swallowing)
			Injections	(i) As it is a painful procedure, the patient feels uncomfortable during injection
			Direct section of the nerve in the cerebellopontine angle	(i) High morbidity with neurologic and life threatening condition (ii) Thromboembolic complication (iii) Meningitis (iv) Cerebrospinal fluid leak, (v) Cutaneous flap distension (vi) Facial nerve dysfunction (vii) Ocular dysfunction (viii) Tinnitus
		Sedatives (on condition)	Microvascular decompression	(i) Low recurrence of pain (ii) Chance of nerve damage result (iii) Hoarseness (iv) Difficulty swallowing (dysphagia) (v) Unsteady gait

**Table 4:** Radiological criteria in the diagnosis of TN, MPDS, and GPN.

Diseases	Imaging (CT/MRI)
TN	Vascular compression of the trigeminal nerve (root entry zone) (demyelination and remyelination)
	MS plaque (dorsal root entry zone)
	Trigeminal ganglion (degenerative hypermyelination and microneuromata)

MPDS	Condyle and disc position of TMJ

GPN	Vascular compression of the glossopharyngeal nerve (root entry zone)

## Abbreviations

TN: Trigeminal neuralgia
MPDS: Myofascial pain dysfunction syndrome
GPN: Glossopharyngeal neuralgia
MS: Multiple sclerosis
TrPs: Trigger points
TMJ: Temporomandibular joint
TMD: Temporomandibular disorders
MRI: Magnetic resonance imaging
CT scan: Computed tomography scanning
IASP: International Association for the Study of Pain
IHS: International Headache Society
MVD: Microvascular decompression.
